# Mitochondrial DNA mutations in Medulloblastoma

**DOI:** 10.1186/s40478-023-01602-0

**Published:** 2023-07-27

**Authors:** Viktoria L. E. Funke, Sarah Sandmann, Viktoria Melcher, Jochen Seggewiss, Judit Horvath, Natalie Jäger, Marcel Kool, David T. W. Jones, Stefan M. Pfister, Till Milde, Stefan Rutkowski, Martin Mynarek, Julian Varghese, Ronald Sträter, Stephan Rust, Anja Seelhöfer, Janine Reunert, Barbara Fiedler, Ulrich Schüller, Thorsten Marquardt, Kornelius Kerl

**Affiliations:** 1 Department of Pediatric Hematology and Oncology, University Children’s Hospital Münster, Albert-Schweitzer-Campus 1, 48149 Münster, Germany; 2 Institute of Medical Informatics, University of Münster, 48149 Münster, Germany; 3 Institute of Human Genetics, University Hospital Münster, Münster, Germany; 4 Hopp Children’s Cancer Center Heidelberg (KiTZ), Heidelberg, Germany; 5 Division of Pediatric Neurooncology, German Cancer Research Center (DKFZ) and German Cancer Consortium (DKTK), Heidelberg, Germany; 6 Princess Máxima Center for Pediatric Oncology, Utrecht, The Netherlands; 7 Division of Pediatric Glioma Research, German Cancer Research Center (DKFZ), Heidelberg, Germany; 8 Department of Pediatric Oncology, Hematology and Immunology, Heidelberg University Hospital, Heidelberg, Germany; 9 National Center for Tumor Diseases (NCT), Heidelberg, Germany; 10 Clinical Cooperation Unit Pediatric Oncology, German Cancer Research Center (DKFZ) and German Consortium for Translational Cancer Research (DKTK), Heidelberg, Germany; 11 Department of Pediatric Hematology and Oncology, University Medical Center Hamburg-Eppendorf, 20251 Hamburg, Germany; 12 Mildred Scheel Cancer Career Center HaTriCS4, University Medical Center Hamburg-Eppendorf, Hamburg, Germany; 13 Department of General Pediatrics, Metabolic Diseases, University Children’s Hospital Münster, 48149 Münster, Germany; 14 Department of Neuropediatrics, University Children’s Hospital, Münster, Germany; 15 Research Institute Children’s Cancer Center, 20251 Hamburg, Germany; 16 Institute of Neuropathology, University Medical Center Hamburg-Eppendorf, 20251 Hamburg, Germany

**Keywords:** Medulloblastoma, DNA, Mitochondrial, Mitochondrial diseases, DNA mutational analysis

## Abstract

To date, several studies on genomic events underlying medulloblastoma (MB) biology have expanded our understanding of this tumour entity and led to its division into four groups—WNT, SHH, group 3 (G3) and group 4 (G4). However, there is little information about the relevance of pathogenic mitochondrial DNA (mtDNA) mutations and their consequences across these. In this report, we describe the case of a female patient with MB and a mitochondriopathy, followed by a study of mtDNA variants in MB groups. After being diagnosed with G4 MB, the index patient was treated in line with the HIT 2000 protocol with no indications of relapse after five years. Long-term side effects of treatment were complemented by additional neurological symptoms and elevated lactate levels ten years later, resulting in suspected mitochondrial disease. This was confirmed by identifying a mutation in the *MT-TS1* gene which appeared homoplasmic in patient tissue and heteroplasmic in the patient’s mother. Motivated by this case, we explored mtDNA mutations across 444 patients from ICGC and HIT cohorts. While there was no statistically significant enrichment of mutations in one MB group, both cohorts encompassed a small group of patients harbouring potentially deleterious mtDNA variants. The case presented here highlights the possible similarities between sequelae caused by MB treatment and neurological symptoms of mitochondrial dysfunction, which may apply to patients across all MB groups. In the context of the current advances in characterising and interpreting mtDNA aberrations, recognising affected patients could enhance our future knowledge regarding the mutations’ impact on carcinogenesis and cancer treatment.

**Supplementary Information:**

The online version contains supplementary material available at 10.1186/s40478-023-01602-0.

## Introduction

Although mitochondrial DNA (mtDNA) mutations have been reported in numerous malignancies, in most cases, the pathogenic role of these variants has yet to be clarified [4]. This uncertainty is likely due to the various phenotypes of mitochondrial dysfunction. The consequences of one mtDNA variant can, for example, vary depending on the organ affected and the heteroplasmy level. It is often assumed that variants exceeding 60% to 80% heteroplasmy have a biochemical effect [9]. Nonetheless, for susceptible organs like the brain, even variants beneath this threshold might result in disabilities such as vision and hearing loss, epilepsy, ataxia, and cognitive impairment [8, 9]. This diversity poses a challenge in predicting the impact of variants. At the same time, it seems particularly intriguing, as it might contribute to gradients and stages within tumours [4].

Medulloblastoma (MB) is one of the most frequent primary malignant brain tumour entities in children. Four established MB groups, namely WNT, SHH, group 3 (G3) and group 4 (G4), differ in molecular driver events and patient outcomes [1]. However, there have been only a few investigations of the role of mtDNA variants in this tumour entity, and none of these studies considered an association with the four MB groups [2, 5, 10–12]. Here, we report the case of a female patient suffering from group 4 MB and mitochondriopathy and put this into the context of mtDNA variants in 444 MB patients across two distinct cohorts.

## Case presentation

The index patient was first diagnosed with G4 MB at the age of seven. After complete resection of the tumour, fractionated craniospinal radiotherapy with simultaneous administration of vincristine followed by chemotherapy (cisplatin, lomustine and vincristine) was conducted according to the HIT 2000 protocol (ClinicalTrials.gov/NCT00303810). Follow-up screenings showed no signs of relapse in MRI after five years. Throughout treatment, the patient suffered from cerebellar mutism and neurological side effects such as ataxia, balance disorders and abducens paresis. Ten years after diagnosis, she presented with clinical signs such as sensorineural hearing loss, fatigue and seizures combined with increased lactate and lactate-pyruvate quotient, indicating a mitochondriopathy. Tissues of the patient and the mother (both: leukocytes, hair, bladder epithelium; patient only: tumour tissue) were tested, and a mutation in the gene encoding the mitochondrial tRNA for serine (*MT-TS1*) was found in all samples. The variant’s heteroplasmy ranged from 20% (leukocytes) to 70% (bladder epithelium) in the patient’s asymptomatic mother but was found almost homoplasmic (heteroplasmy $\ge$ 89%) in patient tissue, indicating segregation with the disease (Additional file 1: Fig. S1). Within the following months, the girl developed therapy-resistant epilepsy with bilateral generalised seizures, progressive ataxia, and dysphagia. Despite all efforts, she died from the sequelae of the mitochondriopathy.

## Cohort study

Based on this case, we raised the question of whether there might be a connection between pathogenic mtDNA variants and MB occurrence or phenotype. We analysed mtDNA variants in MB, employing whole-genome sequencing data of 491 patients from the ICGC cohort and 57 formalin-fixed paraffin-embedded human G3/G4 MB tumour samples of 54 patients from the HIT cohort, explored by mtDNA targeted sequencing. Samples of 101 patients and one additional control sample of the ICGC cohort were excluded from further analysis due to low coverage of the mtDNA. Following the initial calling of 34,072 (ICGC) and 4,224 (HIT) variants, respectively, all calls were annotated and filtered, aiming to identify pathogenic variants (Additional file 1).

**Fig. 1:**
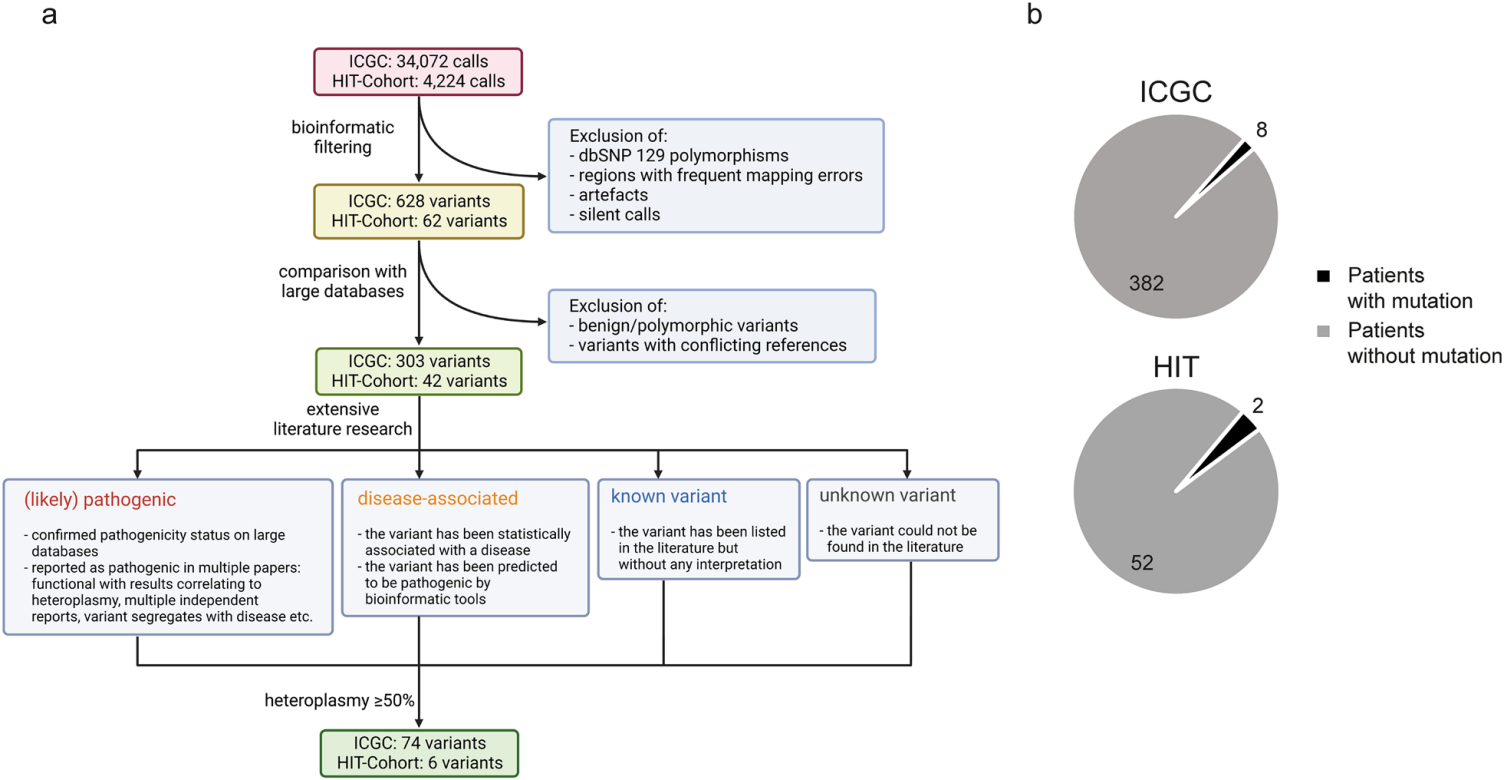
Pathogenic mtDNA variants with high levels of heteroplasmy in MB patients **a** Overview of the variant filtering and classification workflow created with Biorender.com. The numbers shown refer to the number of calls at each analysis stage regardless of the number of patients harbouring these calls. **b** Circle plots illustrating the proportion of patients with and without mtDNA variants in ICGC (top) and HIT (bottom) cohorts. A patient was counted as mutated if an observed variant had been classified as (likely) pathogenic or disease-associated and showed a heteroplasmy of at least 50%

We found 303 mutations in the ICGC and 42 mutations in the HIT cohort (Fig. 1a, Additional file 1: Fig. S2 and S3). In both cohorts, more than 85% of final calls were single nucleotide variants, and most mutations were located in the coding area of the mtDNA. The mean level of heteroplasmy, determined as variant allele frequency (VAF), was 31.78% in ICGC and 18.88% in the HIT cohort (Additional file 1: Fig. S3). To increase the likelihood of examined mutations causing mitochondrial dysfunction, we subsequently focused on variants with at least 50% heteroplasmy (n = 80).

A small group of patients harbouring (likely) pathogenic or disease-associated mutations with high heteroplasmy appeared to be present in both cohorts encompassing eight patients (2.05%) in the ICGC cohort and two patients (3.7%) in the HIT cohort (Fig. 1b, Additional file 1: Fig. S4). However, there was no prevalence for samples with mtDNA mutations to be enriched in one MB group in the ICGC cohort (Additional file 2: Table S1). Referring to the variant detected in the index patient, one pathogenic mutation in *MT-TS1* was detected in both cohorts (Additional file 1: Fig. S3a), exhibiting low heteroplasmy in all patients affected (VAF < 10%).

## Discussion and conclusions

Although several reports have shown associations of mtDNA mutations with various tumour entities [10, 12], only a few studies assessed their highly context-dependent biological consequences in vivo or in vitro [4]. The introduction of cytoplasmic hybrid (cybrid) cells, methods to manipulate a variant’s heteroplasmy, and other models might enable future research to improve variant classification and receive new insights into the role of mitochondrial dysfunction in cancer [3]. In the course of this progress, it has already been demonstrated that mtDNA variants might not only be involved in carcinogenesis but also in cancer progression and the adaptability of tumours towards changing environments [4]. Furthermore, their impact on therapy efficacy and safety is currently under discussion [7]. Therefore, mtDNA aberrations may become relevant to the affected cancer patients in multiple areas of their disease.

Taken together, the case reported here highlights that neurological symptoms of mitochondrial dysfunction can closely resemble long-term sequelae of MB treatment. As demonstrated in the analysis of two cohorts, the consequences of confounding between these two pathogenicities might affect a fraction of patients in all MB groups. It remains to be elucidated whether mtDNA mutations are involved in tumorigenesis or impact tumour cell functions in these patients or a specific subgroup within MB. Nevertheless, against the background of the current advances regarding the functional analysis of mtDNA mutations, identifying the according patients could become important regarding our understanding of tumour formation, progression, and treatment outcomes.

## Supplementary Information

**Additional file 1**. Supplementary materials include supplementary methods, results, supplementary **Figures S1–S4**, and references.

**Additional file 2. Table S1**. Results of Fisher’s exact tests comparing MB groups in the ICGC cohort are shown regarding an enrichment of samples harbouring a mutation in general and an enrichment of mutations in one area of the mtDNA. For both versions, statistical analysis was conducted one time considering all variants, regardless of heteroplasmy, and one time including only variants with at least 50% heteroplasmy. Adjustment of *p*-values was conducted using Bonferroni correction. *MB* Medulloblastoma, *G3* Group 3, *G4* Group 4, *CDS* Coding sequence.

## Data Availability

Whole-genome sequencing data of 491 patients from the ICGC cohort were previously published by Northcott et al. [6].

## Abbreviations

ACMG: American college of medical genetics and genomics
CDS: Coding sequence
FFPE: Formalin-fixed paraffin-embedded
G3: Group 3
G4: Group 4
IGV: Integrated genome viewer
INDEL: Insertion and/or deletion
MB: Medulloblastoma
mtDNA: Mitochondrial DNA
mut: Mutated
Position in MT: Position on the mitochondrial DNA
SNV: Single nucleotide variant
VAF: Variant allele frequency
VUS: Variant of unknown significance
wt: Wildtype
