# Supplementary material for: Mitochondrial DNA mutations in Medulloblastoma
Source: Acta Neuropathol Commun. 2023 Jul 27;11:124. doi: 10.1186/s40478-023-01602-0 (PMC10373251; doi:10.1186/s40478-023-01602-0)
Supplement: Supplementary file 1 — Additional file 1. Supplementary materials include supplementary methods, results, supplementary Figures S1–S4, and references. [file 40478_2023_1602_MOESM1_ESM.docx]

**Supplementary materials for**

**Mitochondrial DNA mutations in Medulloblastoma**

Journal: Acta Neuropathologica Communications

Viktoria LE Funke^1^, Sarah Sandmann^2^, Viktoria Melcher^1^, Jochen Seggewiss^3^, Judit Horvath^3^, Natalie Jäger^4,5^, Marcel Kool^4-6^, David TW Jones^4,7^, Stefan M Pfister^4,5,8,9^, Till Milde^4,8-10^, Stefan Rutkowski^11^, Martin Mynarek^11,12^, Julian Varghese^2^, Ronald Sträter^1^, Stephan Rust^13^, Anja Seelhöfer^13^, Janine Reunert^13^, Barbara Fiedler^14^, Ulrich Schüller^11,15,16^, Thorsten Marquardt^13^, Kornelius Kerl^1^

^1^Department of Pediatric Hematology and Oncology, University Children’s Hospital Münster, 48149 Münster, Germany

^2^Institute of Medical Informatics, University of Münster, 48149 Münster, Germany

^3^Institute of Human Genetics, University Hospital Münster, Münster, Germany

^4^Hopp Children's Cancer Center Heidelberg (KiTZ), Heidelberg, Germany

^5^Division of Pediatric Neurooncology, German Cancer Research Center (DKFZ) and German Cancer Consortium (DKTK), Heidelberg, Germany

^6^Princess Máxima Center for Pediatric Oncology, Utrecht, The Netherlands

^7^Division of Pediatric Glioma Research, German Cancer Research Center (DKFZ), Heidelberg, Germany

^8^Department of Pediatric Oncology, Hematology and Immunology, Heidelberg University Hospital, Heidelberg, Germany

^9^National Center for Tumor Diseases (NCT), Heidelberg, Germany

^10^Clinical Cooperation Unit Pediatric Oncology, German Cancer Research Center (DKFZ) and German Consortium for Translational Cancer Research (DKTK), Heidelberg, Germany

^11^Department of Pediatric Hematology and Oncology, University Medical Center Hamburg-Eppendorf, 20251 Hamburg, Germany

^12^Mildred Scheel Cancer Career Center HaTriCS4, University Medical Center Hamburg-Eppendorf, Hamburg, Germany

^13^Department of General Pediatrics, Metabolic Diseases, University Children's Hospital Münster, 48149 Münster, Germany

^14^Department of Neuropediatrics, University Children’s Hospital, Münster, Germany

^15^Research Institute Children's Cancer Center, 20251 Hamburg, Germany

^16^Institute of Neuropathology, University Medical Center Hamburg-Eppendorf, 20251 Hamburg, Germany

**Corresponding author**

Kornelius Kerl, M.D., Ph.D.

Email: kornelius.kerl@ukmuenster.de

**Table of Contents**

[1. Supplementary methods 3](#_Toc125547448)

[1.1. mtDNA sequencing and variant calling 3](#_Toc125547449)

[1.2. Classification and filtering of mtDNA variants 3](#_Toc125547450)

[1.3. Statistical analysis 5](#_Toc125547451)

[2. Supplementary results 6](#_Toc125547452)

[3. Supplementary figures 7](#_Toc125547453)

[4. Supplementary references 10](#_Toc125547454)

# **1. Supplementary methods**

## **1.1. mtDNA sequencing and variant calling**

Within the scope of this study, mitochondrial DNA (mtDNA) variants of 444 MB patients were examined using whole-genome sequencing data of 491 patients from the ICGC cohort (matching tumour and control samples of 490 patients) and 57 formalin-fixed paraffin-embedded (FFPE) human G3/G4 MB samples of 54 patients from German HIT cohort. The index patient described in the main text was not part of these cohorts. For the HIT cohort, targeted sequencing of mtDNA was conducted according to the TWIST NGS workflow (Twist Bioscience, San Francisco, CA, USA) with the TWIST mitochondrial panel (#102039). Quality control was carried out utilising Agilent High Sensitivity D5000 gDNA Tapestation Assay. For sequencing MiniSeq High Output Reagent kit (150 cycles) from Illumina (#FC-420-1002) was employed. All bioinformatic analyses in the following were conducted utilising R version 4.0.3 [9]. Alignment against the reference genome GRCh37 was performed using BWA mem [6]. Tumour and control samples of 101 patients and one additional control sample from the ICGC cohort were excluded from subsequent analyses as they exhibited only low or intermediate coverage results (<80% min 100x cov). Variant calling was performed using appreci8 [12] (default settings: minimum read depth 50, minimum number of variant allele reads 20) with adapted filtering for mtDNA (see section 1.2). High coverage of remaining samples enabled confident variant detection at low heteroplasmy levels (variant allele frequency >1%). One variant exhibiting a lower heteroplasmy (VAF = 0.98%) in the tumour sample was included as this variant’s heteroplasmy exceeded 1% in the matching control sample.

## **1.2. Classification and filtering of mtDNA variants**

After initial mutation calling, quality-filtered variant calls were annotated utilising mtSNP, mtDB, ClinVar, 1000Genomes and Cosmic [1, 3, 5, 13, 14] and filtered based on call characteristics and annotation information aiming for pathogenic variants. Additionally, a manual investigation of all borderline calls was performed using the integrated genome viewer (IGV). After removing artefacts, polymorphisms and regions with frequent mapping errors [4], variants were compared to large databases of mtDNA variants and underwent extensive literature research to be grouped into the classes suggested by the American College of Medical Genetics and Genomics (ACMG): benign, likely benign, variants of unknown significance (VUS), likely pathogenic, and pathogenic [11]. As the ACMG guidelines mainly refer to variants of the nuclear genome and do not take characteristics specific to the mtDNA into account, grouping was performed based on recommended criteria for mtDNA variant classification [15, 17–19] (Fig. 1a, Additional file 1: Fig. S2). To give a more detailed insight into variant characteristics, the large group of VUS were divided into four groups: disease-associated, known, unknown, and variants with conflicting references. Disease-associated variants, for example, did not fulfil all criteria to be classified as pathogenic or likely pathogenic but had been statistically associated with a disease or predicted to be deleterious before. Subsequently, calls classified as benign, likely benign or conflicting were excluded leaving 303 variants in ICGC and 42 variants in the HIT cohort.

All databases used within the scope of this analysis are listed below.

| Database | Link | Accession Date/Version |
| --- | --- | --- |
| Mitomap (Mitomaster) | https://www.mitomap.org/MITOMAP | 01.03.2021 |
| GenBank | Via Mitomaster | - |
| HelixMtdb | https://www.helix.com/pages/mitochondrial-variant-database | 24.02.2021 |
| mtSNP | http://mtsnp.tmig.or.jp/mtsnp/index_e.shtml | 20.01.2021 |
| mtDB | http://www.mtdb.igp.uu.se/ | 20.01.2021 |
| ClinVar | https://www.ncbi.nlm.nih.gov/clinvar/ | clinvar_20190916, and  common_no_known_medical_  impact_20160203 |
| 1000Genomes | https://www.internationalgenome.org/ | Phase 3 |
| Cosmic | https://cancer.sanger.ac.uk/cosmic | 76 |
| Haplogrep2 | https://haplogrep.i-med.ac.at/category/haplogrep2/ | 08.04.2021 |
| Wong et al. 2020 | DOI: 10.1038/s41436-019-0746-0  DOI: 10.1002/humu.24082 | - |
| Yuan et al. 2020 | DOI: 10.1038/s41588-019-0557-x | - |
| Triska et al. 2019 | DOI: 10.1158/0008-5472.CAN-18-2220 | - |

## **1.3. Statistical analysis**

Statistical analysis was conducted using R version 4.1.3 [10]. Fisher’s exact test was performed to compare the number of samples containing mtDNA mutations between all four MB groups in the ICGC cohort. Analysis was carried out one time considering all final 303 variants of this cohort after the exclusion of benign and conflicting calls and another time focussing on variants classified as pathogenic, likely pathogenic or disease-associated. For the latter version, it was also tested whether samples with mutations in one specific area of the mtDNA (mRNA, tRNA, rRNA) would be enriched in one group. This test was performed twice as one sample harboured both tRNA and mRNA variants. All analytic steps were repeated, considering only variants with at least 50% heteroplasmy. As the variants of the sample harbouring both tRNA and mRNA mutations did not exceed this level of heteroplasmy, testing for the enrichment of variants in one specific area of the mtDNA was only performed once this time. The resulting p-values were adjusted for multiple testing performing Bonferroni correction.

The statistical analysis was not performed for the HIT cohort, as it consisted solely of G3/G4 MB.

# **2. Supplementary results**

Pathogenic or disease-associated mutations with high heteroplasmy (VAF ≥50%) were detected in both cohorts (ICGC: 8 patients; HIT: 2 patients; Fig. 1b). Clinical characteristics of the patients from the ICGC cohort harbouring these mutations are shown in Additional file 1: Fig. S4. All eight patients from the ICGC cohort and one of two patients from the HIT cohort harboured pathogenic or diseases associated variants close to homoplasmy (VAF $\geq$90%, Additional file 1: Fig. S3) similar to the variant of the index patient. Strikingly, when it comes to the ICGC cohort, these calls were all germline variants observed in tumour and control tissue.

One pathogenic mutation in *MT-TS1*, which harboured the mutation in the index patient, was found in both cohorts (ICGC: 2xSHH, 1xG3, 1xG4; 1 HIT-sample) (Additional file 1: Fig. S3). M.7465insC is located next to a poly-C stretch causing the same genomic sequence as m.7471insC. The latter has been reported pathogenic in various databases and large-scale studies with experimental evidence for causing mitochondrial malfunction [7, 18].

# **3. Supplementary figures**


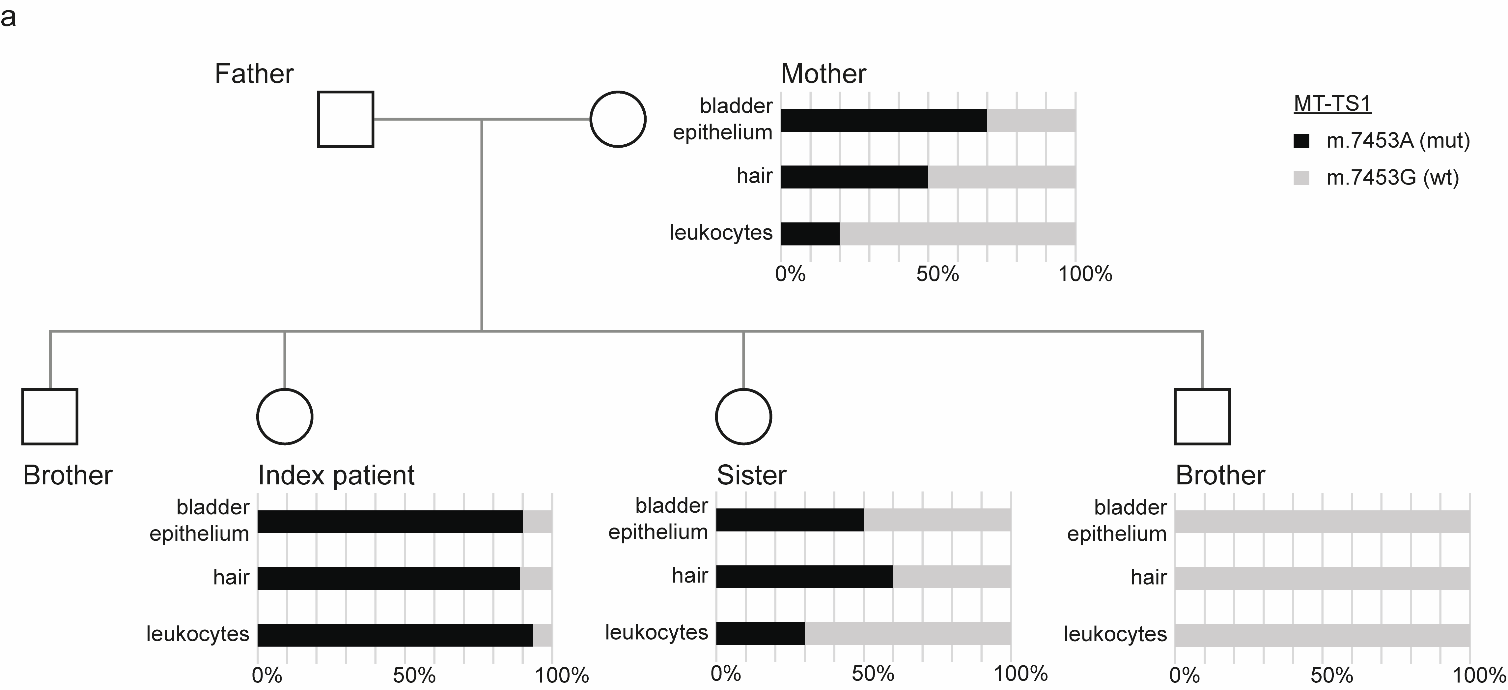


**Fig. S1 (related to the case report) Levels of heteroplasmy across family members of the index patient**

a) Ancestral tree depicting varying levels of heteroplasmy of the variant affecting the index patient across different family members and tissues. Mutational information about the patient’s father and one brother were not available. mut = mutated; wt = wildtype


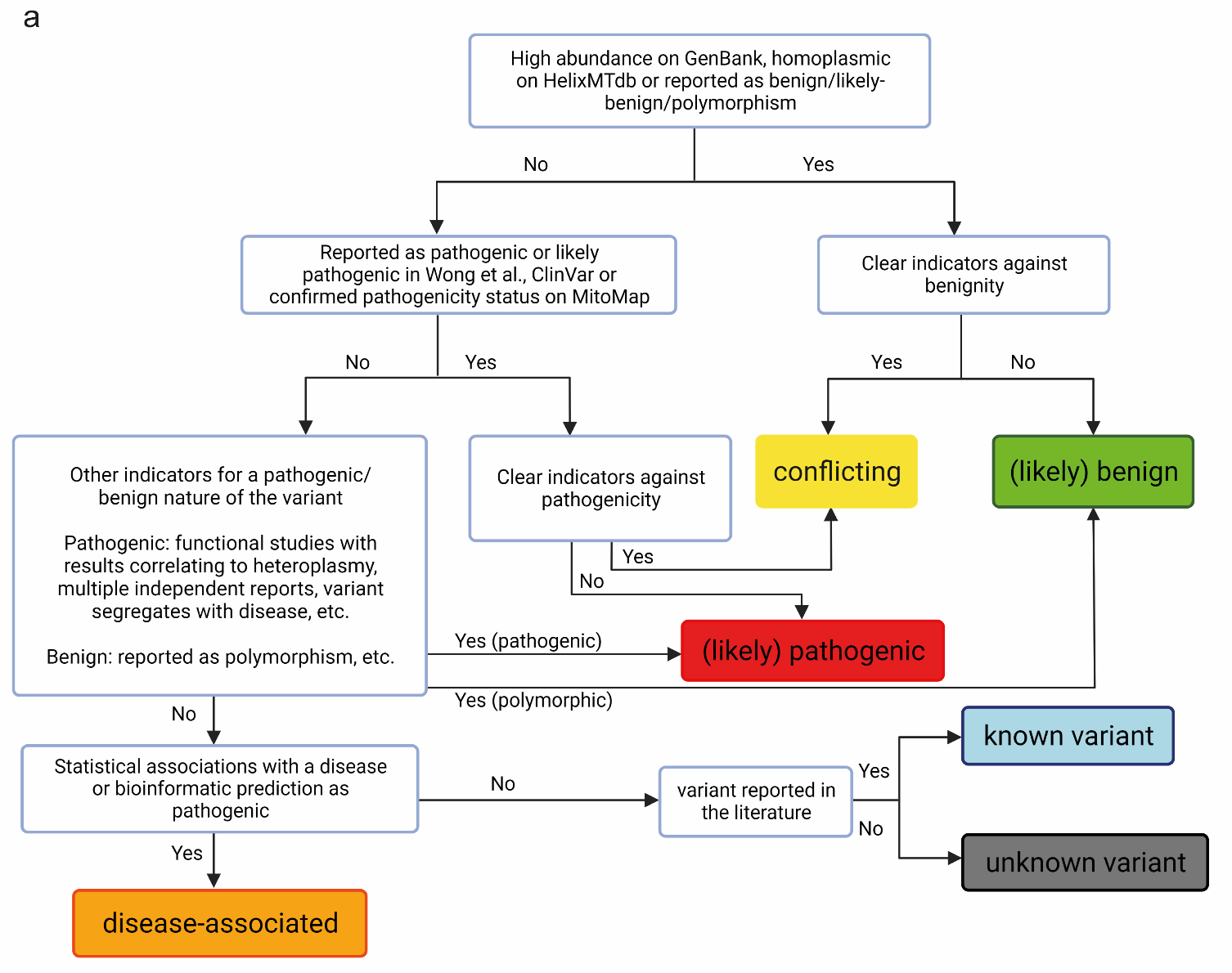


**Fig. S2 (related to Fig. 1) Classification of mtDNA variants**

a) A flowchart providing more details on variant classification based on recommended criteria for mtDNA mutations is shown. The category VUS has been divided into the groups: disease-associated, known variants, unknown variants, and variants with conflicting references. In the first tile of the graphic, high abundance has been defined as a variant which occurred homoplasmic in more than 100 cases on HelixMTdb [2]. Every call without any other information on its pathogenicity found in the literature and less than ten homoplasmic reports on HelixMTdb was classified as a “known variant” or “unknown variant”. This graphic was created using Biorender.com.


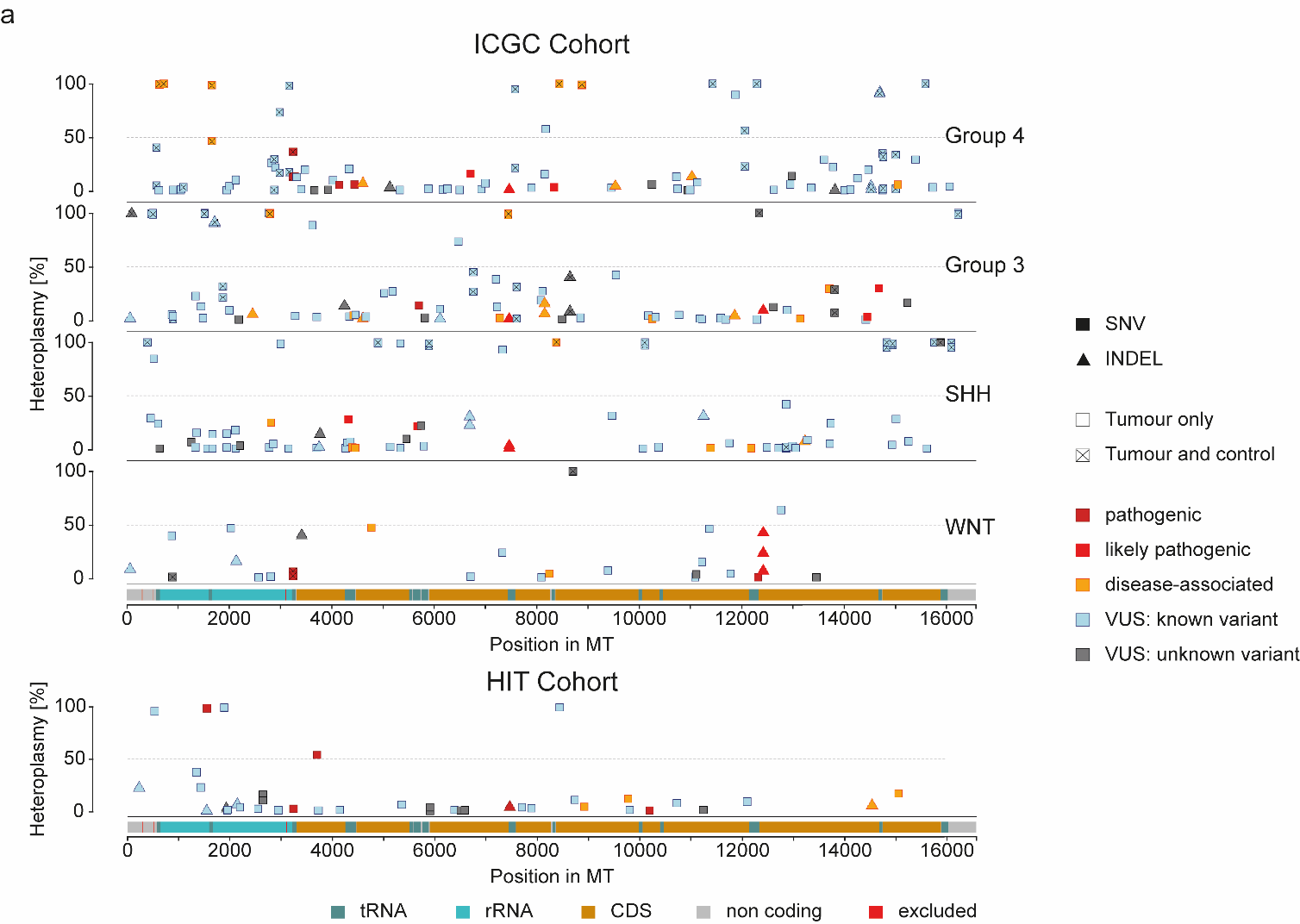


**Fig. S3 (related to Fig. 1) Distribution of mtDNA variants across MB groups and the mitochondrial genome**

a) Plot shows all final variants after filtering, as outlined in Fig. 1a). The x-axis refers to the position of calls on the mitochondrial DNA and the y-axis to the heteroplasmy level. SNVs are represented by squares, and INDELs by triangles. Variants which occurred in the tumour and control tissue have been highlighted with a cross. Colours indicate the variant classification as shown. For the ICGC cohort, calls have been separated depending on the MB group affiliation of the mutated sample. Pathogenic, likely pathogenic, and disease-associated variants exceeding a heteroplasmy of 50%, as indicated by dotted lines, have been considered for creating circle plots shown in Fig. 1b). SNV = single nucleotide variant; INDEL = insertion and/or deletion; VUS = variant of unknown significance; Position in MT = Position on the mitochondrial DNA; CDS = coding sequence


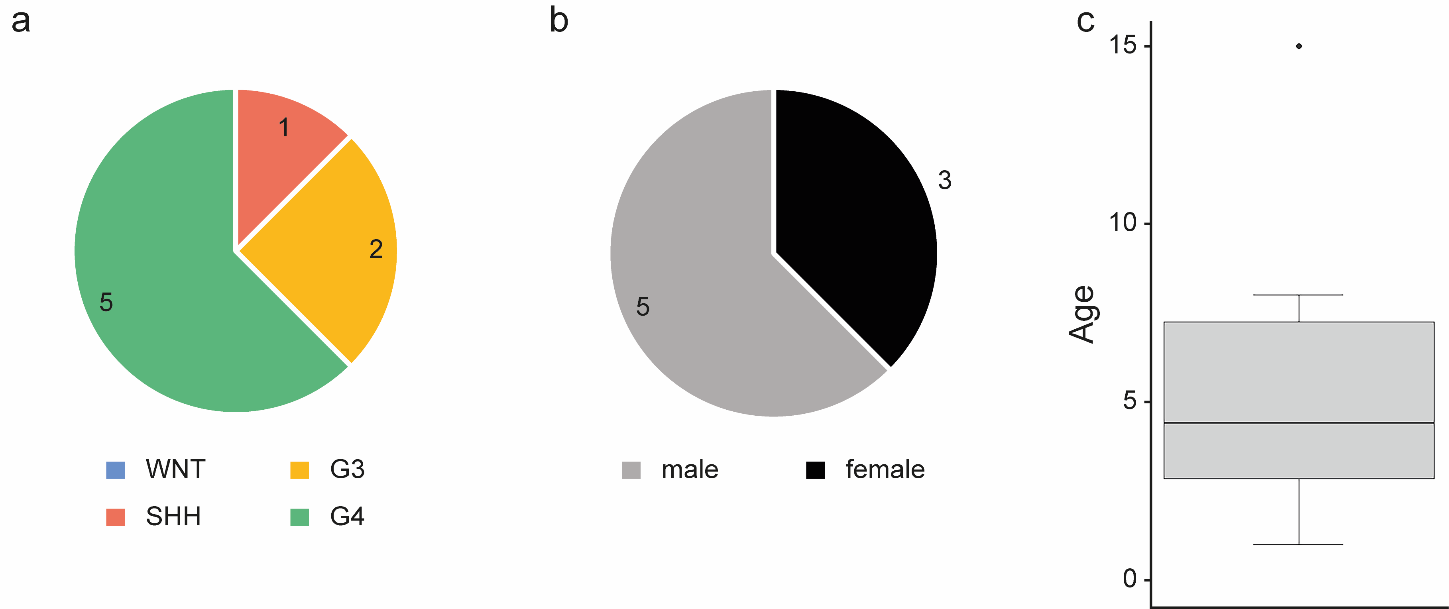


**Fig. S4 (related to Fig. 1) Clinical characteristics of patients from the ICGC cohort with disease-associated mtDNA variants with high heteroplasmy**

Eight patients from the ICGC cohort harboured disease-associated mtDNA variants with high levels of heteroplasmy. Clinical characteristics of these patients derived from Northcott et al. [8], including a) MB group, b) gender, and c) age distribution, are shown. Boxplot was created using ggplot2 [16] and R version 4.1.3 [10].

# **4. Supplementary references**

1. The 1000 Genomes Project Consortium (2015) A global reference for human genetic variation. Nature 526:68–74. doi: 10.1038/nature15393
2. Bolze A, Mendez F, White S, Tanudjaja F, Isaksson M, Jiang R, et al (2020) A catalog of homoplasmic and heteroplasmic mitochondrial DNA variants in humans. bioRxiv. doi: 10.1101/798264
3. Ingman M, Gyllensten U (2006) mtDB: Human Mitochondrial Genome Database, a resource for population genetics and medical sciences. Nucleic Acids Res 34:D749–D751. doi: 10.1093/nar/gkj010
4. Ju YS, Alexandrov LB, Gerstung M, Martincorena I, Nik-Zainal S, Ramakrishna M, et al (2014) Origins and functional consequences of somatic mitochondrial DNA mutations in human cancer. Elife 3:e02935. doi: 10.7554/eLife.02935
5. Landrum MJ, Lee JM, Benson M, Brown GR, Chao C, Chitipiralla S, et al (2018) ClinVar: improving access to variant interpretations and supporting evidence. Nucleic Acids Res 46:D1062–D1067. doi: 10.1093/nar/gkx1153
6. Li H (2013) Aligning sequence reads, clone sequences and assembly contigs with BWA-MEM. arXiv:1303.3997
7. Lott MT, Leipzig JN, Derbeneva O, Xie HM, Chalkia D, Sarmady M, et al (2013) mtDNA Variation and Analysis Using Mitomap and Mitomaster. Curr Protoc Bioinformatics 44:1.23.1-26. doi: 10.1002/0471250953.bi0123s44
8. Northcott PA, Buchhalter I, Morrissy AS, Hovestadt V, Weischenfeldt J, Ehrenberger T, et al (2017) The whole-genome landscape of medulloblastoma subtypes. Nature 547(7663):311-317. doi: 10.1038/nature22973
9. R Core Team (2020) R: A Language and Environment for Statistical Computing. R Foundation for Statistical Computing, Vienna, Austria. https://www.R-project.org/
10. R Core Team (2022) R: A Language and Environment for Statistical Computing. R Foundation for Statistical Computing, Vienna, Austria. https://www.R-project.org/
11. Richards S, Aziz N, Bale S, Bick D, Das S, Gastier-Foster J, et al (2015) Standards and guidelines for the interpretation of sequence variants: a joint consensus recommendation of the American College of Medical Genetics and Genomics and the Association for Molecular Pathology. Genet Med 17:405–424. doi: 10.1038/gim.2015.30
12. Sandmann S, Karimi M, de Graaf AO, Rohde C, Göllner S, Varghese J, et al (2018) appreci8: a pipeline for precise variant calling integrating 8 tools. Bioinformatics 34:4205–4212. doi: 10.1093/bioinformatics/bty518
13. Tanaka M, Cabrera VM, González AM, Larruga JM, Takeyasu T, Fuku N, et al (2004) Mitochondrial Genome Variation in Eastern Asia and the Peopling of Japan. Genome Res 14:1832–1850. doi: 10.1101/gr.2286304
14. Tate JG, Bamford S, Jubb HC, Sondka Z, Beare DM, Bindal N, et al (2019) COSMIC: the Catalogue Of Somatic Mutations In Cancer. Nucleic Acids Res 47:D941–D947. doi: 10.1093/nar/gky1015
15. Wang J, Schmitt ES, Landsverk ML, Zhang VW, Li FY, Graham BH, et al (2012) An integrated approach for classifying mitochondrial DNA variants: one clinical diagnostic laboratory’s experience. Genet Med 14:620–626. doi: 10.1038/gim.2012.4
16. Wickham H (2016) ggplot2: Elegant Graphics for Data Analysis. Springer-Verlag New York
17. Wong LC, Chen T, Schmitt ES, Wang J, Tang S, Landsverk M, et al (2020) Clinical and laboratory interpretation of mitochondrial mRNA variants. Hum Mutat 41:1783–1796. doi: 10.1002/humu.24082
18. Wong LC, Chen T, Wang J, Tang S, Schmitt ES, Landsverk M, et al (2020) Interpretation of mitochondrial tRNA variants. Genet Med 22:917–926. doi: 10.1038/s41436-019-0746-0
19. Yarham JW, Elson JL, Blakely EL, McFarland R, Taylor RW (2010) Mitochondrial tRNA mutations and disease. Wiley Interdiscip Rev RNA 1:304–324. doi: 10.1002/wrna.27
